# The brief pain inventory—Interference Subscale has acceptable reliability but questionable validity in acute back and neck pain populations

**DOI:** 10.1016/j.bjpt.2024.101150

**Published:** 2024-11-21

**Authors:** Caitlin M.P. Jones, Chung-Wei Christine Lin, Joshua Zadro, Arianne Verhagen, Mark Hancock, Raymond Ostelo

**Affiliations:** a Sydney Musculoskeletal Health, The University of Sydney, The Institute for Musculoskeletal Health, Sydney Local Health District, Level 10 N KGV Building, Missenden Rd, Camperdown 2050, Sydney, NSW, Australia; b Graduate School of Health, University of Technology Sydney, Sydney, NSW, Australia; c Faculty of Medicine, Health and Human Sciences, Macquarie University Sydney, Sydney, NSW, Australia; d Amsterdam Movement Sciences Research Institute, Department of Health Sciences, Vrije University, Amsterdam, North Holland, the Netherlands; e Department of Epidemiology and Data Science, Amsterdam UMC, Vrije Universiteit, Amsterdam, North Holland, the Netherlands

**Keywords:** Back pain, Brief pain inventory, Clinimetric, Functional impairment, Interference subscale, Neck pain

## Abstract

**Background:**

The Brief Pain Inventory—Interference Subscale (BPI-IS) is a subscale of the BPI assessment tool developed to rapidly assess the impact of a person's pain on their function. It is uncertain whether it has one or two factors, and whether it has acceptable clinimetric properties in a mixed spinal pain (back and/or neck) population.

**Objectives:**

To determine the clinimetric properties of the BPI-IS in a population with mixed spinal pain.

**Methods:**

We completed a clinimetric evaluation with a test-retest design, factor analysis, and hypothesis testing. We used data collected for a randomised clinical trial including a population presenting to primary care or emergency departments with acute spinal pain (back and/or neck).

**Results:**

Confirmatory factor analysis better supported the two-factor model of the BPI-IS (physical interference factor and affective interference factor) as compared to the one-factor model. Both one and two-factor models had acceptable reliability (high internal consistency and no evidence of floor or ceiling effects). Both models failed to reach our a-priori thresholds for acceptable construct (cross sectional) validity, and responsiveness (longitudinal validity) in either back or neck pain populations.

**Conclusion:**

The BPI-IS has two factors and both have acceptable reliability, but tests for validity did not reach our a priori thresholds for acceptability (construct validity and responsiveness). The BPI-IS may not be suitable to measure the impact of pain on function in back and neck pain populations.

## Highlights

• In a mixed spinal pain population, the BPI-IS has two factors of interest.
• Clinicians and researchers should use the BPI-IS with caution in this population.
• The tool has acceptable reliability, but we could not confirm validity.

## Introduction

The Brief Pain Inventory-Interference Subscale (BPI-IS) is a subscale of the BPI assessment tool developed to rapidly assess the impact of a person's pain on their function.^1^ The BPI was originally developed for use with people with cancer pain, however it has been tested in a broad range of pain populations such as osteoarthritis,^2^ chronic non-malignant pain,^3^ post-operative pain,^4^ and neuropathic pain^5^ and found to have acceptable clinimetric properties. The developers recommend that the BPI-IS be used as a unifactor tool, with scores reported as the average of the 7 total BPI-IS items. However, there is some uncertainty in more recent literature about whether the BPI-IS subscale should be treated as a one factor model (interference) or a two-factor model (physical interference and affective interference). Multiple studies have reported that both models are equally appropriate in both acute and chronic mixed pain samples.^6^, ^7^, ^8^ To investigate the tool's construct validity, it first needs to be confirmed whether it is measuring one factor or two in this specific population with acute spinal pain.^9^ Understanding whether the tool has one or two factors will also guide researchers and clinicians as to how to interpret the results (e.g. if a person scores poorly on items from the physical factor but not the affective factor, conclusions and interventions can be tailored).

Spinal pain is the leading cause of disability worldwide and disability is often assessed with condition specific tools such as the Roland-Morris Disability Questionnaire^10^ for back pain and the Neck Disability Index^11^ for neck pain. These tools have acceptable clinimetric properties; however, they have some limitations. The BPI-IS is a generic tool and can potentially be used in populations that have either (or both) back and neck pain. This would be useful in settings such as clinical trials and practice where there are populations of mixed spinal pain, and it would avoid the need to use separate tools for those with neck and back pain, which decreases the power of the trial to detect a difference in this outcome (by splitting sample sizes up by specific area of pain).

The BPI has two subscales, pain severity and pain interference. The pain severity subscale has been previously examined in spinal pain populations,^12^ however the interference subscale (BPI-IS) has not. The BPI (both subscales) was used as an outcome measure in a recent placebo-controlled trial of opioid analgesia for acute spinal pain (the OPAL trial),^13^ which presented an opportunity to examine the clinimetric properties of this subscale.

We aimed to investigate whether the BPI-IS subscale has one or two factors to inform future use of the tool in research and practice. We also aimed to examine the reliability (internal consistency) and validity (cross-sectional construct validity and responsiveness) of the BPI-IS in a mixed spinal (back and/or neck) pain population.

## Methods

### Design

This clinimetric study uses data from the OPAL trial^13^ (registration number ACTRN 12615000775516), approved by the University of Sydney Human Research Ethics Committee (approval number 2015/004). All participants consented to participate in the research. The OPAL trial recruited 347 participants with acute back and/or neck pain and collected pain and disability measures at baseline, randomized them to receive either an opioid or an identical placebo, and performed 6 follow-up time-points over 12-months. This is a secondary analysis of the OPAL trial (ACTRN 12615000775516) using the timepoints baseline and 6-weeks. A sample size of 50 has been suggested as ‘adequate’ for clinimetric studies to assess construct validity, reliability, floor/ceiling effects, and ‘very good’ to assess responsiveness, which this study has surpassed overall and for both subgroups (back pain and neck pain).^14^

### Study population

Participants included in this study are people who presented to either a hospital emergency department or general practitioner with acute low back and/or neck pain (with or without distal radiation). Their pain was at least of moderate severity (measured by adaptations of item 7 of the SF-36) and ≤12 weeks (acute). People with reoccurring pain needed to have had at least one month of being pain-free prior to this episode to be included. Exclusion criteria included known or suspected serious pathology (e.g. infection, cauda equina syndrome, metastatic disease), contraindications to opioid analgesics, having taken a prescription opioid for the current episode of pain, and having had spinal surgery in the previous six months.

### Outcome measures

Participants completed the BPI, the Roland Morris Disability Questionnaire (RMDQ) if they had low back pain and the Neck Disability Index (NDI) if they had neck pain, and the Short Form 12 Item Health Survey (SF-12v2) at baseline over the phone with a researcher.

Six weeks after baseline, participants repeated the above measures, as well as the Global Perceived Effect scale. These were completed mainly online, or on the phone with a researcher (the same researcher who conducted the baseline assessment in most cases). A summary of each outcome measure used in the present clinimetric study is below.

The BPI is a tool with two subscales, one to measure pain intensity and the other to measure pain interference, and has been validated within many populations, although with varying reports of measurement error.^15^ The tool's user guide instructs that the interference subscale should be scored as the average of all 7-items, e.g. as a one-factor tool, although it does acknowledge provisional evidence that there may be two factors (physical interference and affective interference) within the subscale. There is partial evidence for the convergent validity of the whole BPI tool (both subscales) in the context of back pain. One study found a moderate correlation of 0.66 between BPI and Oswestry Disability Index scores.^16^ A recent review of the BPI Pain Intensity subscale found partial evidence for construct validity in the context of back pain, however, they did not examine the Interference subscale.^12^ This was completed by all participants.

The RMDQ is a tool that measures disability (another word for pain related function) in people with back pain.^17^ Validity testing has shown good construct validity compared to other tools,^18^ restricted content validity due to the specificity of the tool's scope,^19^ and unclear responsiveness.^18^ Reliability testing of the RMDQ has shown high internal consistency (Cronbach's alpha of 0.92),^20^ high test-retest reliability (intraclass correlation coefficient of 0.91),^17^ but uncertain measurement error.^18^ Responsiveness (longitudinal validity) testing of the RMDQ has shown a correlation of 0.49 with the Global Perceived Effect Scale (GPE scale).^21^ This was completed by participants who had back pain (including those who had back and neck pain).

The NDI^11^ is a commonly used tool to measure disability in people with neck pain. It has also been found to have desirable clinimetric properties which make it a suitable choice for measuring disability associated with neck pain, such as internal consistency (Cronbach's alpha of 0.87 to 0.92) and convergent construct validity (moderate to strong correlations with other valid instruments measuring pain and function), but may have inadequate test-retest reliability (intraclass correlation coefficient of 0.5).^22^ This was completed by participants who had neck pain (including those who had back and neck pain).

The SF-12v2^23^ measures 8 domains of general health-related wellbeing. It has been shown to have acceptable construct and criterion validity (moderate to strong correlations with perceived health) and internal consistency (Cronbach's alpha 0.85) and test-re rest reliability (intraclass correlation coefficient of 0.72) in non-cancer pain populations.^24^ This was completed by all participants.

The GPE scale is a measure of a person's opinion of how their current state compares to a previous time point. In the OPAL study, participants were presented with an 11-point continuous scale of −5 (vastly worse) to +5 (vastly better) with zero (no change) as the mid-point. These types of scales have been designed to be clinically relevant in all populations due to the open nature of the question allowing the patient to consider factors as broadly or narrowly as they see as appropriate to their current state.^25^^,^^26^ This was completed by all participants.

### Study procedures

Confirmatory factor analysis using Jamovi 1.6.23 software was performed to test whether a one-factor or two-factor model had a better fit for this sample. The two factors are ‘physical’ (general activity, walking ability, and normal work) and ‘affective’ (mood, relationships with others, sleep, and enjoyment of life). All reliability, validity, and responsiveness procedures described below were performed on both a one-factor and a two-factor model to investigate whether one shows superior clinimetric properties.

For reliability, the following parameters were considered acceptable based on literature and other similar studies;^7^^,^^27^^,^^28^ a Comparative fit index (CFI) of >0.95, Root-mean-squared error of approximation (RMSEA) of <0.08, Tucker Lewis Index (TLI) of >0.90, and a non-significant Chi Squared test with a *p*-value of >0.05. We reported the standardised factor loading on each factor. We ran this analysis on both back and neck pain populations separately. We also ran this analysis on both back and neck pain populations together, and we investigated one and two-factor models separately. Those with both back and neck pain were included in both analyses. We considered a Cronbach's alpha of >0.70 as acceptable internal consistency.^29^

Floor and ceiling effects for each model (one factor versus two) in each population (back and neck) were evaluated by assessing what percentage of participants scored either the highest or lowest score. Floor and ceiling effects were considered present if ≥15 % of participants achieved the highest or lowest score at baseline. We did not assess floor and ceiling effects at 6-weeks as a large proportion of the population had recovered by that time point (no longer had acute back and/or neck pain).

Construct (cross sectional) validity was assessed using the method of hypothesis testing, as described in the COSMIN checklist,^30^ and done in a similar study.^31^ The construct of the BPI-IS that this study aimed to investigate is pain related disability in patients with at least moderate acute low back and/or neck pain. For the one-factor model, we generated 11 hypotheses, initially determined based on literature review and then refined based on consensus by a panel of experts (MH and AV). For the two-factor model, we generated 22 hypotheses (11 for each of the two factors of the BPI-IS—physical and affective). Hypotheses based on correlations were graded with the following definitions: Pearson's r of >0.90 = very strong, Pearson's r of 0.70–0.90 = strong, Pearson's r of 0.50–0.69 = moderate, Pearson's r of 0.30–0.49 = weak, and Pearson's r of <0.29 = not correlated. We considered it reasonable to accept the construct validity of each of the factors of the BPI-IS if >75 % of hypotheses can be proven.^30^

Responsiveness was assessed by correlating the change scores between baseline and 6 weeks of the BPI-IS and the GPE. We did this separately for each model (one or two-factors). We considered at least a moderate correlation (Pearson's r of 0.50 or higher) to be an acceptable threshold for responsiveness.

## Results

### Participant characteristics

Out of 347 participants, 331 completed the BPI-IS assessment at baseline. Twelve participants had their BPI-IS missing completely, and four had partially missing data. There were 308 participants with back pain, and 68 people with neck pain. These figures both include 30 people with both back and neck pain. One person did not specify the location of their pain and did not complete any assessment. Our sample of 335 participants (347 excluding 12 with completely missing BPI-IS data) included 170 males (51 %) and 165 females (49 %). Mean age was 45 years with a range of 18 to 90 years. The mean pain intensity at baseline was 22.6 on a 0 to 40 scale of the BPI Pain Intensity subscale.

### Internal consistency

Confirmatory factor analysis was performed on all available data (including partially missing participant data; *n* = 335). We found evidence that is more supportive of a two-factor model of the BPI-IS for back pain, and there was no clear difference for neck pain.

For back pain, including those with both back and neck pain (*n* = 308), a one-factor model had the following; CFI = 0.93, TLI = 0.89, RMSEA = 0.11, and Chi^2^ test significance of <0.001 (Supplementary material 1.1). The two-factor model had the following: CFI = 0.98, TLI = 0.97, RMSEA = 0.06, and Chi^2^ significance of 0.027 (Supplementary material 1.2). The one-factor model did not meet all the thresholds for acceptability. All measures in the two-factor model met the a priori thresholds for acceptability except for the Chi^2^ test (which was not met in either case).

For neck pain, including those with both neck and back pain (*n* = 68), a one-factor model had the following; CFI = 0.99, TLI = 0.99, RMSEA = 0.04, Chi^2^ insignificance of *p* = 0.334 (Supplementary material 1.3).

A two-factor model had the following: CFI = 0.99, TLI = 0.99, RMSEA = 0.04, Chi^2^ insignificance of *p* = 0.364, (Supplementary material 1.4). All measures met the a priori thresholds for acceptability and were very similar between the two models.

For mixed spinal pain (the entire sample; *n* = 335), a one-factor model had the following; CFI = 0.91, TLI = 0.86, RMSEA = 0.12 and Chi^2^ test significance of <0.001 (Supplementary material 1.5). The two-factor model had the following: CFI = 0.98, TLI = 0.97, RMSEA = 0.06, and Chi^2^ significance of 0.006 (Supplementary material 1.6). The one-factor model did not meet all the thresholds for acceptability. All measures in the two-factor model met the a priori thresholds for acceptability except for the Chi^2^ test (which was not met in either case).

Cronbach's alpha showed acceptable internal consistency in both models, with each factor in each population meeting our a priori range of ≥0.70 but <0.95 (Table 1).

**Table 1:** Internal consistency measured by Cronbach's alpha on a one and two-factor model.

One factor model		
Population	One pain interference factor	
Back or both	0.83	
Neck or both	0.86	
Mixed spinal pain (entire sample)	0.83	
Two factor model		
Population	Physical factor	Affective factor
Back or both	0.76	0.76
Neck or both	0.77	0.78
Mixed spinal pain (entire sample)	0.78	0.77

### Floor and ceiling effects

To assess floor and ceiling effects we used data from 335 participants who either partially or fully completed their baseline assessment. As all proportions are less than 15 % (Table 2, Table 3), there is no evidence of floor or ceiling effects in either the one or two-factor model of the BPI-IS in the back, neck, or both pain groups.

**Table 2:** Floor and ceiling effects in a one-factor model.

Effect	Pain interference factor
Back pain (*n* = 268)	
Floor	1 (0.3 %)
Ceiling	1 (0.3 %)
Neck pain (*n* = 38)	
Floor	0 (0.0 %)
Ceiling	0 (0.0 %)
Both back and neck pain (*n* = 29)	
Floor	0 (0.0 %)
Ceiling	0 (0.0 %)
Mixed spinal pain (entire sample) (*n* = 335)	
Floor	1 (0.3 %)
Ceiling	1 (0.3 %)

**Table 3:** Floor and ceiling effects in a two-factor model.

Effect	Physical interference factor		Affective interference factor
Back pain (*n* = 268)			
Floor		2 (0.7 %)	4 (1.5 %)
Ceiling		12 (4.5 %)	2 (0.8 %)
Neck pain (*n* = 38)			
Floor		2 (5.2 %)	0 (0.0 %)
Ceiling		1 (2.6 %)	1 (2.6 %)
Both back and neck pain (*n* = 29)			
Floor		0 (0.0 %)	0 (0.0 %)
Ceiling		1 (3.4 %)	0 (0.0 %)
Mixed spinal pain (all participants combined; *n* = 335)			
Floor		4 (1.0 %)	4 (1.0 %)
Ceiling		14 (4.1 %)	3 (0.9 %)

### Construct (cross-sectional)

For the one-factor model, only 4 of the 11 hypotheses (36 %) were confirmed in the participants with back pain, and 6 of the 11 (54 %) were confirmed in the participants with neck pain. Therefore, these data did not support an acceptable construct validity for the BPI-IS in these populations (hypotheses and results table in Supplementary material 2).

For the two-factor model, only 14 of the 22 hypotheses (7 out of 11 for each factor) (64 %) were confirmed. Therefore, these data did not support an acceptable construct validity for the BPI-IS in these populations (Supplementary material 3).

For responsiveness, in total, there were 269 participants with both a baseline and 6-week BPI-IS score, and 240 also had a GPE score at 6 weeks. For the one-factor model, the correlation between BPI-IS change scores and GPE at six weeks was weak (−0.35 for back, neck, and mixed pain locations; Table 4). For the two-factor model the correlation between BPI-IS change scores and GPE at six weeks was weak for physical factor (−0.37 for back, neck, and combined pain locations) and for the affective factor (−0.48 for back, neck, and combined pain locations; Table 5). Neither model reached our a priori hypothesis of moderate correlation. When split by pain location, all correlations were weak (Supplementary material 4). As all groups reported improvement (on average) in their BPI-IS score (demonstrated by all change scores being negative), we conducted a sensitivity analysis on the one-factor model by removing 6 participants who are suspected to have made an error in their surveys. Three participants who selected ‘Much worse’ and 3 who selected ‘Vastly worse’ on the GPE questionnaire had also selected scores on the BPI-IS of 0–1 in all 7 questions, indicating little to no impact of pain on function. We suspected that these 6 participants misunderstood the direction of the scale. With those 6 participant's data removed the correlation coefficient increased but remained ‘weak’ (−0.46).

**Table 4:** GPE and BPI-IS change scores for the one-factor model.

GPE	n	Mean (SD) BPI-IS change score (range is −70 to +70)
Completely recovered (+5)	47	−37.3 (16.5)
Much improved (+4)	52	−30.8 (13.3)
Moderately improved (+3)	31	−21.5 (15.1)
A little improved (+2)	17	−14.2 (21.5)
Slightly improved (+1)	19	−18.7 (16.7)
Unchanged (0)	49	−16.1 (17.4)
Slightly worse (−1)	2	−8.5 (3.5)
A little worse (−2)	6	−33.4 (29.6)
Moderately worse (−3)	5	−11.4 (23.9)
Much worse (−4)	6	−16.8 (30.9)
Vastly worse (−5)	6	−20.3 (26.2)

BPI-IS, Brief Pain Inventory—Interference Subscale; GPE, Global Perceived Effect Scale.

**Table 5:** GPE and BPI-IS change scores for the two-factor model.

GPE	n	Mean (SD) BPI-IS physical factor change score (range is −30 to +30)	Mean (SD) BPI-IS affective factor change score (range is −40 to +40)
Completely recovered (+5)	47	−18.8 (7.3)	−18.5 (10.5)
Much improved (+4)	52	−14.7 (7.4)	−16.1 (8.3)
Moderately improved (+3)	31	−10.7 (6.6)	−10.8 (9.7)
A little improved (+2)	17	−8.1 (8.9)	−8.5 (11.6)
Slightly improved (+1)	19	−8.3 (7.5)	−10 (10.8)
Unchanged (0)	49	−8.6 (8.9)	−7.6 (9.9)
Slightly worse (−1)	2	−5.0 (1.4)	−3.5 (2.1)
A little worse (−2)	6	−9 (15.1)	−13 (16.2)
Moderately worse (−3)	5	−1.2 (9.4)	−10.2 (15.6)
Much worse (−4)	6	−9.5 (13.9)	−7.3 (17.7)
Vastly worse (−5)	6	−10.3 (12.1)	−10.0 (14.4)

BPI-IS, Brief Pain Inventory—Interference Subscale; GPE, Global Perceived Effect Scale.

## Discussion

We tested the clinimetric properties of the BPI-IS in a population with mixed spinal pain (acute back and/or neck pain). Our confirmatory factor analysis supports a two-factor model over a one-factor model for back pain and mixed spinal pain, and found that the models were equivalent for neck pain. The Chi^2^ analysis did not reach the threshold in any case. However, there is a known limitation of the Chi^2^ analysis in confirmatory factor analysis where it can report significant *p*-values when sample sizes exceed 200, even when the difference is trivial.^32^ This is likely the case given that all the other measures are indicating a good fit. The reliability and validity results did not differ substantially between the two models across back, neck, and mixed spinal pain populations. All factors of the BPI-IS have acceptable reliability (high internal consistency and no evidence of floor or ceiling effects) across back, neck, and combined pain groups. The BPI-IS did not have acceptable validity in either back, neck, or combined pain populations, as all models failed to reach our a priori threshold for construct (cross sectional) validity and responsiveness. We did not find substantial differences between the clinimetric properties of the tool between the two populations (back and neck pain). Responsiveness of this tool was weak in all analyses. The BPI-IS has acceptable reliability but questionable validity with regards to measurement of pain-related disability in patients with acute spinal pain (back and/or neck). The BPI-IS may in fact be measuring a different construct to pain-related disability.

To our knowledge, no other study has examined the clinimetric properties of the BPI-IS specifically in a spinal pain population. One other study has examined the entire BPI (pain severity and pain interference) in osteoarthritis and low back pain^33^ and found acceptable reliability. However, they found evidence of acceptable validity of a one-factor model for the BPI-IS, which is different from our findings. One possible explanation for this is chronicity of the included populations. Osteoarthritis is a chronic condition, and while the chronicity of the back pain subgroup was not specified, it is possible they also had lasting conditions compared to our sample, which was specifically acute pain. Also, the authors assessed validity by analysing correlations between the BPI-IS as a one-factor model and two or three other pain or disability measurement tools, rather than a hypothesis testing method as we have done here (with 11 a-priori formulated hypotheses per factor).

Limitations of this study include a small sample size for the neck pain group. While we reached the threshold of > 50 participants for a clinimetric study of this nature, 30 of the 68 neck pain participants also had back pain. Therefore, there is some uncertainty about the generalisability of these findings to groups with neck pain only. Another potential limitation is that the range used for hypothesis testing were agreed upon by experts, but the numerical cut-offs were somewhat arbitrary, and it is possible that our a priori hypotheses were too optimistic. Another limitation is that our analysis of responsiveness relies on a correlation with the GPE scale, may reflect ‘current’ status rather than change, especially over long timeframes of months.^26^ Our follow up timepoint was 6 weeks, and so while correlating with the GPE is appropriate, the results should be interpreted with caution.

Future research should consider not solely using the BPI-IS to measure pain-related disability for populations with a mix of acute neck and back pain. Condition-specific tools may offer better validity. While the RMDQ has shown good validity for back pain, most of the available literature concerns sub-acute and chronic presentations. It is unclear whether the tool is valid for acute back pain or whether it has similar flaws as the BPI-IS. Further studies validating other tools in acute back and neck pain populations should be conducted. Future use of the BPI-IS in back and neck pain populations should consider reporting the scores of the physical and affective factors separately given that a two-factor model was better supported. Reporting a combined score can be difficult to interpret clinically. For example, a combined score of 40/70 could be comprised of a very high score in the physical factor and a very low score in the affective factor, or vice versa. The interpretation of this result, and direction for therapeutic intervention, would likely differ depending on whether physical or affective interference was the issue.

## Conclusion

Factor analysis supported a two-factor model of the BPI-IS for the entire mixed spinal pain population and the back pain subgroup, although both a one and two factor model were equivalent for the neck pain subgroup. We found that the BPI-IS has acceptable reliability (internal consistency, lack of floor or ceiling effects) across back, neck, and mixed pain populations. However, the BPI-IS did not reach our a-priori thresholds for acceptable validity (construct validity and responsiveness) in any population. The tool should be used with caution if their aim is to measure pain-related disability at any given time point, or to monitor change in pain-related disability over time.

## Conflicts of interest

The authors declare no competing interest.

## Appendix Supplementary materials

Image, application 1
