# Supplementary material for: The brief pain inventory—Interference Subscale has acceptable reliability but questionable validity in acute back and neck pain populations
Source: Braz J Phys Ther. 2024 Nov 21;28(6):101150. doi: 10.1016/j.bjpt.2024.101150 (PMC11616560; doi:10.1016/j.bjpt.2024.101150)
Supplement: Supplementary file 1 [file mmc1.pdf]

Supplementary online material

1.1 – Path diagram for back pain assuming a one-factor model with factor loadings (standardised estimates)

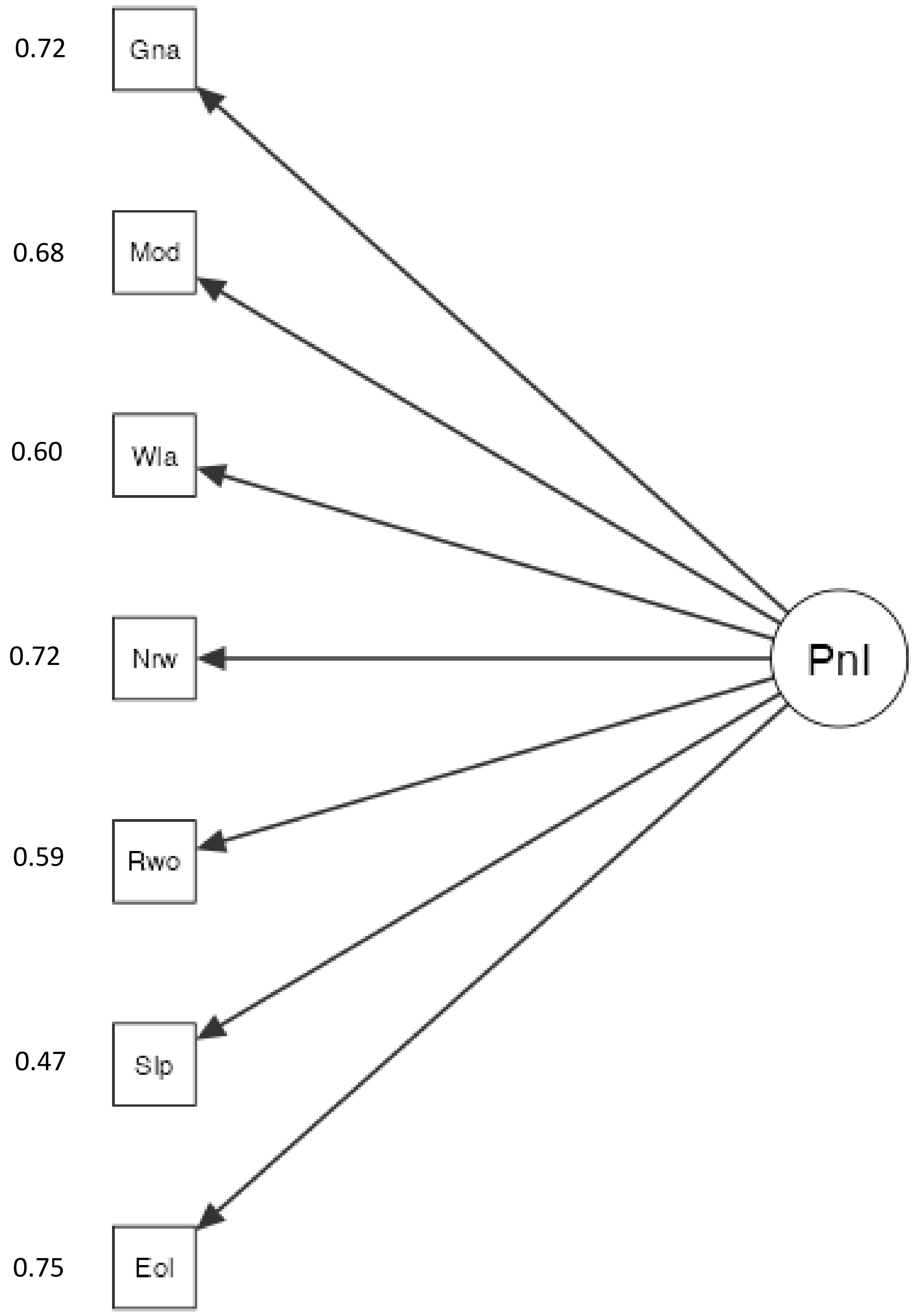

Eol, enjoyment of life; Gna, general activity; mod, mood; Nrw, normal work; PnI, Pain Interference; Rwo, relationships with others; Slp, sleep; Wla, walking ability

1.2 – Path diagram for back pain population assuming a two-factor model with factor loadings (standardised estimates)

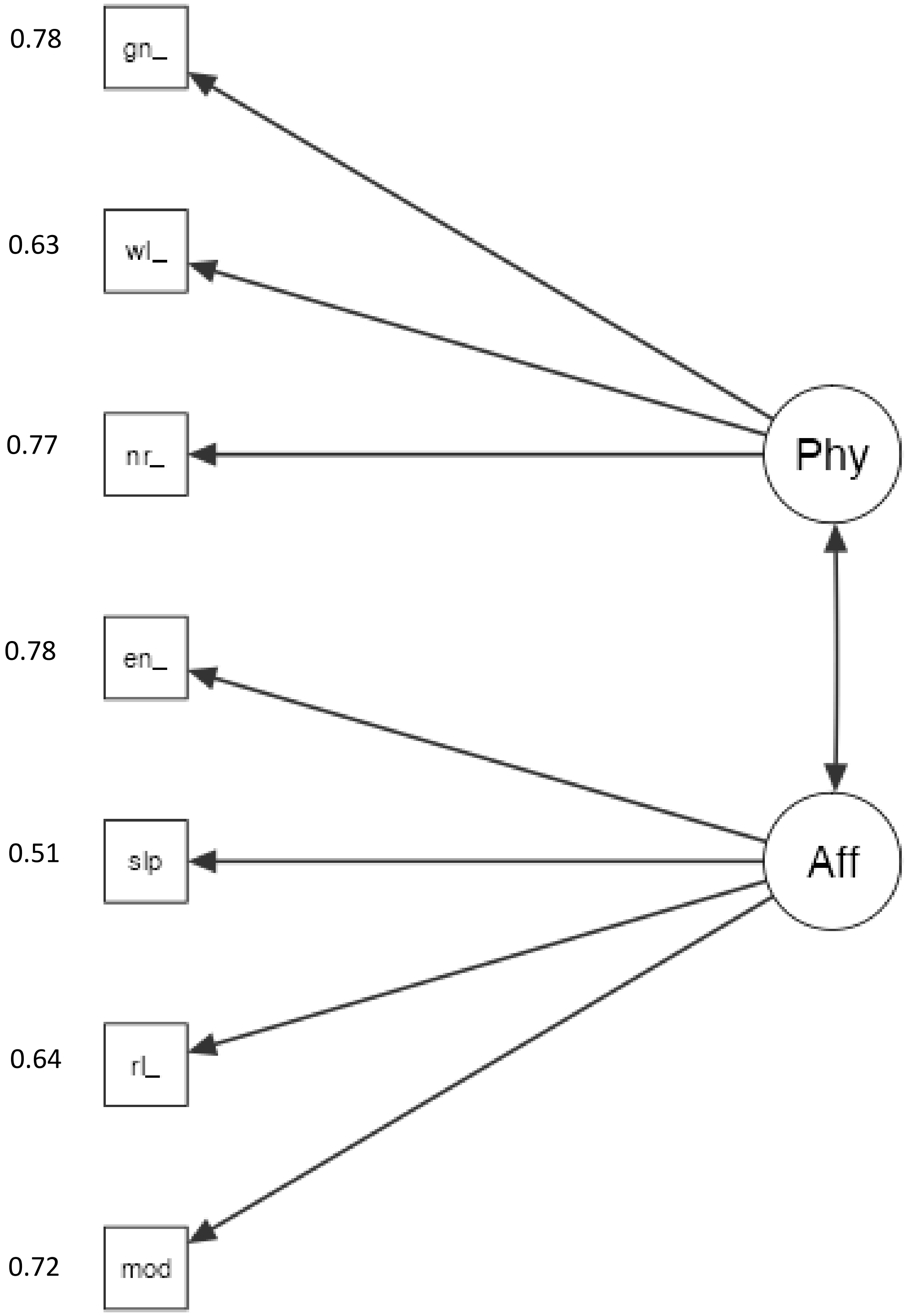

en, enjoyment of life; gn, general activity; mod, mood; nr, normal work; rl, relationships with others; slp, sleep; wl, walking ability

1.3 – Path diagram for neck pain population assuming a one-factor model with factor loadings

(standardised estimates)

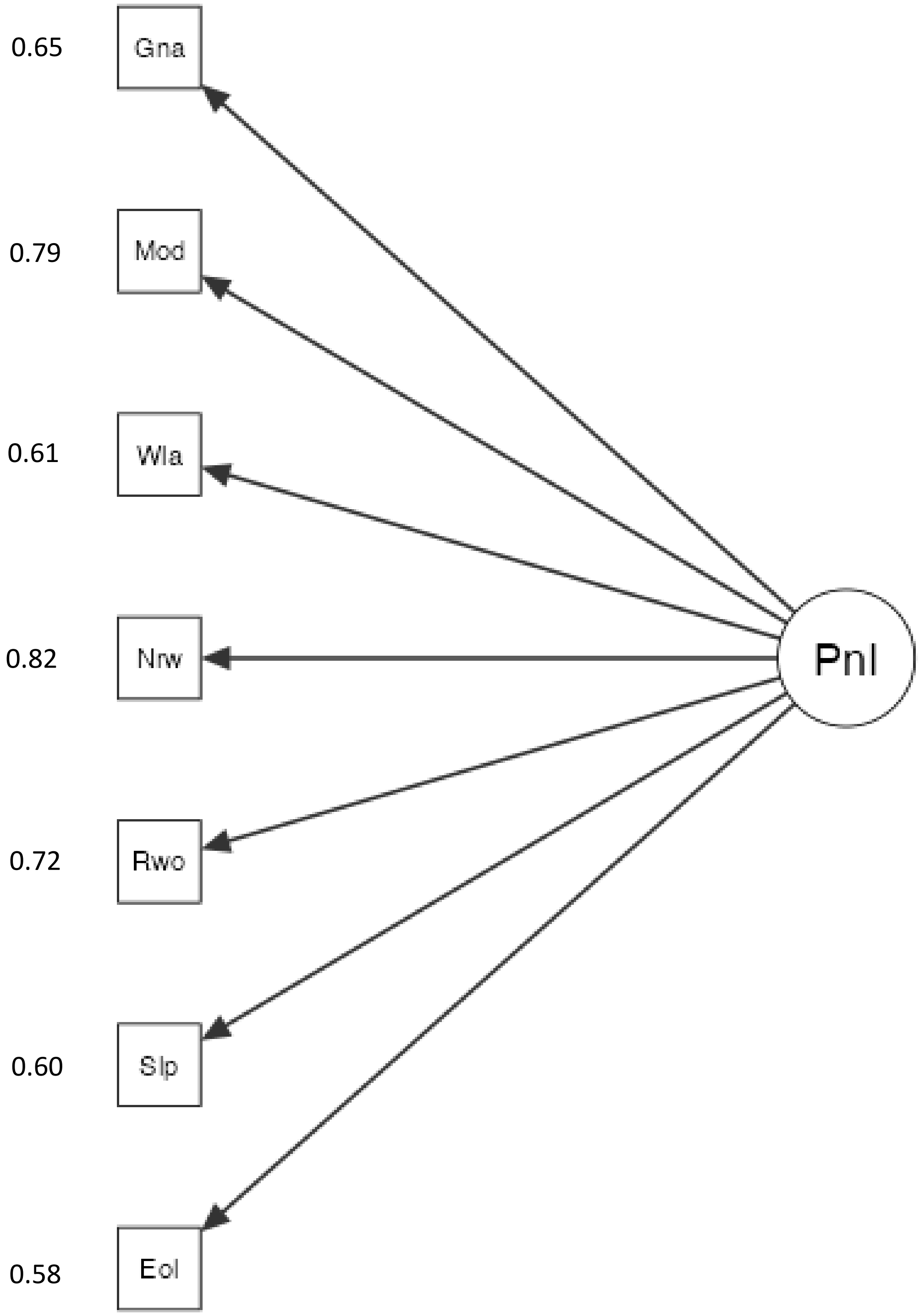

Eol, enjoyment of life; Gna, general activity; mod, mood; Nrw, normal work; PnI, Pain Interference; Rwo, relationships with others; Slp, sleep; Wla, walking ability

1.4 – Path diagram for neck pain population assuming a two-factor model with factor loadings (standardised estimates)

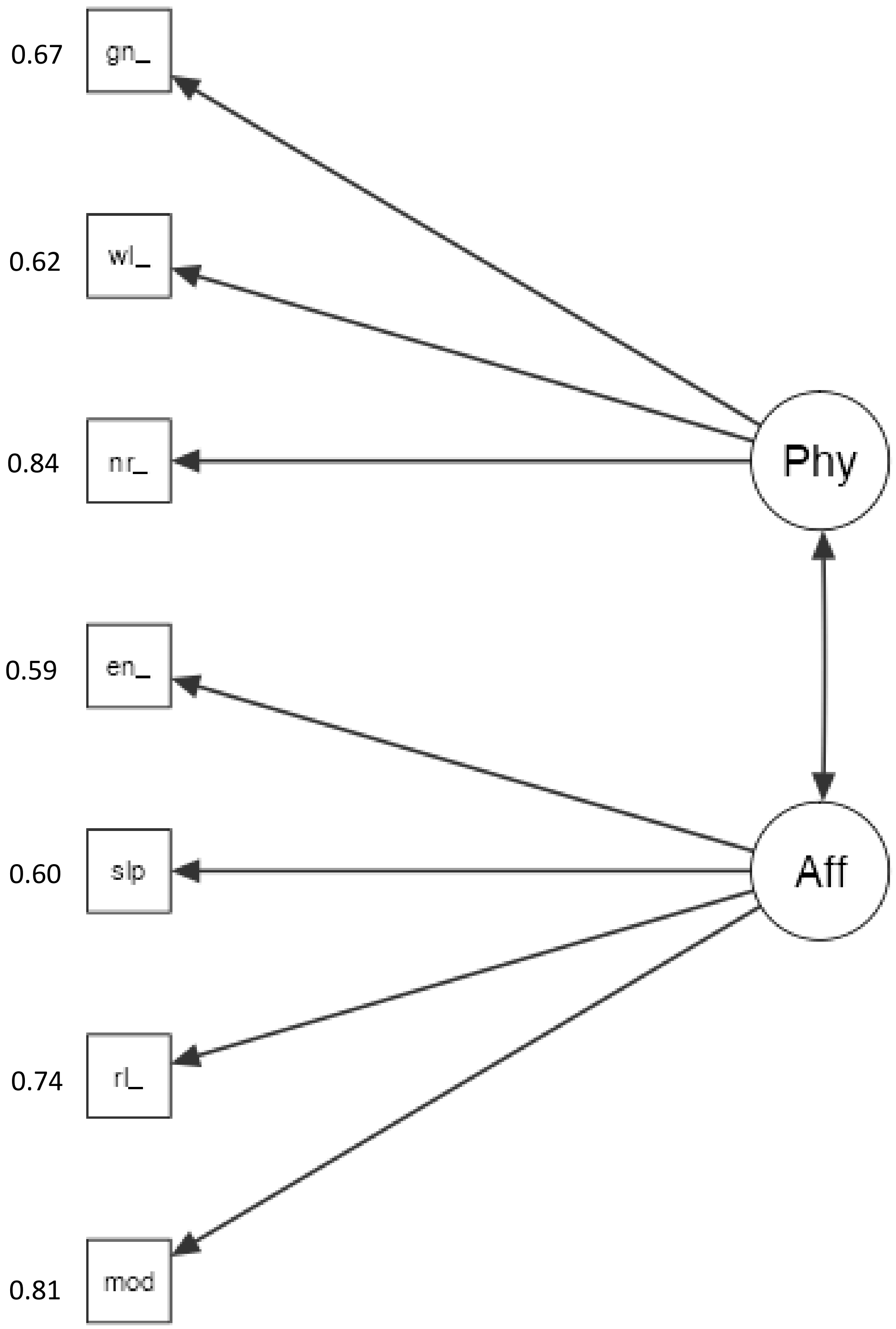

en, enjoyment of life; gn, general activity; mod, mood; nr, normal work; rl, relationships with others; slp, sleep; wl, walking ability

1.5 – Path diagram for mixed spinal pain population assuming a one-factor model with factor loadings (standardised estimates)

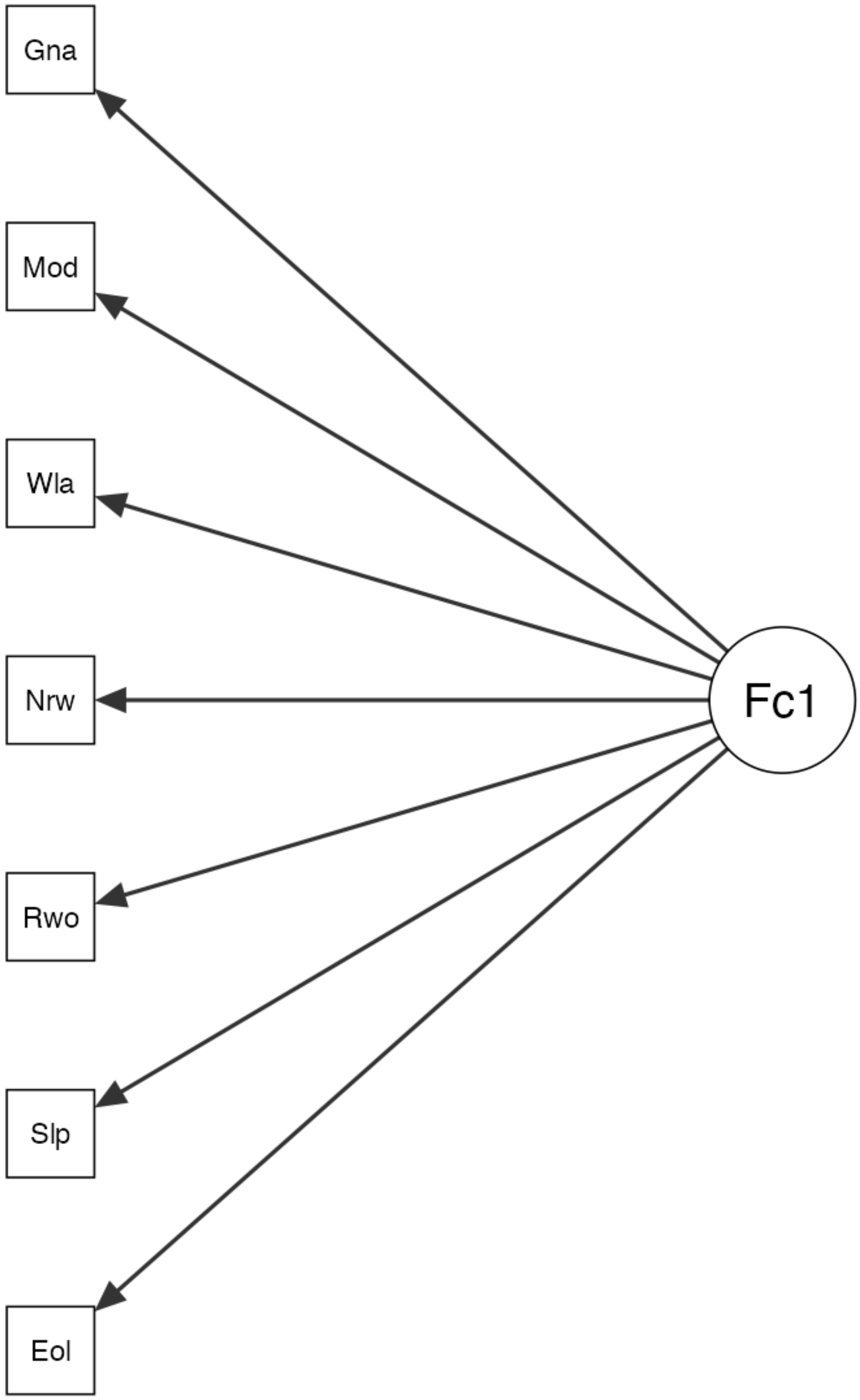

EoI, enjoyment of life; Gna, general activity; Mod, mood; Nrw, normal work; Rwo, relationships with others; Slp, sleep; Wla, walking ability

1.6 – Path diagram for mixed spinal pain population assuming a two-factor model with factor loadings (standardised estimates)

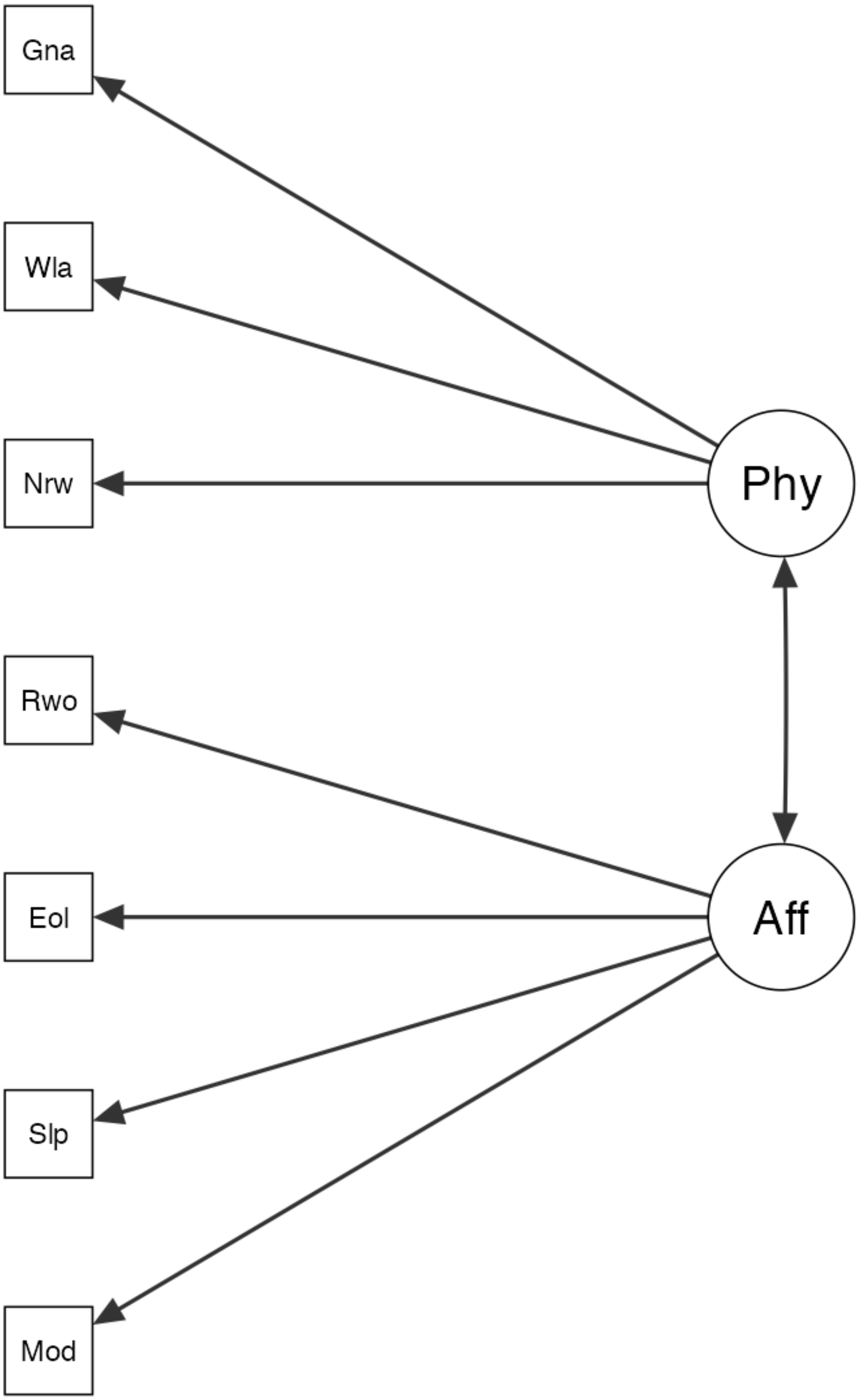

EoI, enjoyment of life; Gna, general activity; Mod mood; Nrw normal work; Rwo, relationships with others; Slp, sleep; Wla, walking ability, , ,

2 - Hypotheses for evaluating construct validity of a one-factor BPI-IS model

**Hypothesis**	**Justification and links to literature**	**Statistical Analysis**	**Result**	**Confirmed: Y / N**
1. The BPI-IS will have at least a moderate positive correlation (Pearson's r of 0.50 to 0.69) with the RMDQ in those with LBP.	The BPI-IS measures a similar but not identical construct to the RMDQ (interference from pain versus disability) therefore a moderate correlation is expected. This is supported by literature which has found the BPI-IS correlates highly with the RMDQ (r=0.81) in a population with osteoarthritis and general non-cancer pain (non-acute).[34,35]	Pearson's correlation coefficient to test convergent validity.	0.62	Y
2. The BPI-IS will have at least a moderate positive correlation (Pearson's r of 0.50 to	The BPI-IS measures a similar but not identical construct to the NDI (interference from pain	Pearson's correlation coefficient to test	0.66	Y

Hypothesis	Justification and links to literature	Statistical Analysis	Result	Confirmed: Y / N
0.69) with the NDI in those with neck pain.	versus disability from pain) therefore a moderate correlation is expected.<br>The NDI has been shown to correlate moderately with other PROMs measuring physical function/disability such as work and lifting in neck pain populations.[36]	convergent validity.		
3. The BPI-IS will have at least a weak positive correlation (Pearson's r of 0.30 to 0.49) with the SF-12v2 (Part 1) e.g. as SF-12v2 score increase (worse general health), BPI-IS	Part 1 asks about general health. General health is a different construct to activity interference from pain however it is sensible to assume that the two constructs are	Pearson's correlation coefficient to test convergent validity.	Back:<br>r = 0.16<br><br>Neck:<br>r = 0.16<br><br>Both combined:	N for both

Hypothesis	Justification and links to literature	Statistical Analysis	Result	Confirmed: Y / N
scores decrease (more activity interference from pain).	somewhat similar, i.e as activity interference from pain reduces, general health improves. Back and neck pain has been found to be associated with general health in a Danish twin study.[37]		r = 0.16	
4. BPI-IS will have at least a strong negative correlation (Pearson's r of -0.70 to -0.90) with the SF-12v2 (Part 2). As SF-12v2 score increase (improving ability to do daily activities), BPI-IS scores decrease (less	Part 2 asks about typical daily activities. This is a fairly generic domain, and theoretically the same domain as the BPI-IS therefore we expect a strong correlation. Both of these tools are not disease specific (unlike the NDI and RMDQ)	Pearson's correlation coefficient to test convergent validity.	Back: r = -0.17<br><br>Neck: r = -0.31<br><br>Both combined: r = -0.16	N for both

Hypothesis	Justification and links to literature	Statistical Analysis	Result	Confirmed: Y / N
activity interference from pain).	which is why this correlation is expected to be higher. This has been demonstrated in literature regarding chronic pain and daily activities [38] and we would expect similar results with acute pain for the duration of the pain episode.			
5. BPI-IS will have at least a moderate negative correlation (Pearson's r of -0.50 to -0.69) with the SF-12v2 (Part 3) e.g. as SF-12v2 score increase (improving ability to accomplish tasks) BPI-	Part 3 asks about limitations of activities in the past week due to physical health, specially accomplishing less and being limited in kinds of activities. This is a similar construct to activity	Pearson's correlation coefficient to measure convergent validity.	Back: r = -0.33<br><br>Neck: r = -0.57<br><br>Both combined: r = -0.34	N for back<br>Y for neck

Hypothesis	Justification and links to literature	Statistical Analysis	Result	Confirmed: Y / N
IS scores decrease (less activity interference from pain).	interference from pain therefore we expect a moderate to strong correlation. No literature is available on this matter to our knowledge.			
6. The BPI-IS will have at least a moderate negative correlation (Pearson's r of -0.50 to -0.69) with the SF-12v2 (Part 5) e.g. as SF-12v2 score increase (improving ability do normal work) BPI-IS scores decrease (less activity interference from pain).	Part 5 asks about how much the pain has interfered with your normal work. This is a similar construct to activity interference from pain therefore we expect a moderate to strong correlation. The AIHW states that back problems are a significant cause of loss of productivity.[39]	Pearson's correlation coefficient to measure convergent validity.	Back: r = -0.42<br>Neck: r = -0.49<br>Both combined: r = -0.41	N for both

Hypothesis	Justification and links to literature	Statistical Analysis	Result	Confirmed: Y / N
7. The BPI-IS will have at least a moderate negative correlation (Pearson's r of -0.50 to -0.69) with the SF-12v2 (Part 6) e.g. as SF-12v2 score increase (improving feelings of peace and energy) BPI-IS scores decrease (less activity interference from pain).	Part 6 asks about how often one feels peaceful and energetic. We consider this domain to be similar to 'depression'. It has been shown to have a moderate correlation with acute pain[40] and a moderate correlation with disability.[41]	Pearson's correlation coefficient to measure convergent validity.	Back: r = -0.34<br><br>Neck: r = -0.30<br><br>Both combined: r = -0.28	N for both
8. The BPI-IS will have at least a moderate positive correlation (Pearson's r of 0.50 to 0.69) with the BPI Pain Severity subscale.	The BPI-IS subscale measures a different construct to the Pain Severity subscale, but it would be sensible to assume the two	Pearson's correlation coefficient to measure convergent validity.	Back: r = 0.44<br><br>Neck: r = 0.58	N for back<br>Y for neck

Hypothesis	Justification and links to literature	Statistical Analysis	Result	Confirmed: Y / N
	correlate to some degree.<br>This is supported by literature examining the relationship between acute pain and disability.[42]		Both combined:<br>r = 0.45	
9. The BPI-IS will have at least a moderate positive correlation (Pearson's r of -0.50 to -0.69) with the VAS pain scale (0-10) (average pain subscale of BPI-PI).	The BPI-IS measures a different construct to pain. However, it is sensible to assume that pain and disability would correlate.<br>This is supported by literature showing a moderate to strong correlation between the BPI-IS and pain scales such as VAS and NRS in chronic LBP.[43]	Pearson's correlation coefficient to measure convergent validity.	Back:<br>r = 0.44<br><br>Neck:<br>r = 0.48<br><br>Both combined:<br>r = 0.40	N for both

Hypothesis	Justification and links to literature	Statistical Analysis	Result	Confirmed: Y / N
10. The BPI-IS will not correlate with the location of a participant's pain (back, neck, or both).	The location of pain (back vs neck) should not correlate with the activity interference from pain.<br>Neither neck pain nor back pain has been demonstrated in literature to be more painful or more activity limiting than the other.	Mean and SDs for each group will be compared.	-0.05	Y
11. The BPI-IS will not correlate with sex.	There is no literature that suggests females experience more disability due to pain. Therefore, we expect similar distributions of scores within sex groups experiencing back and neck pain.	Mean and SDs for each group will be compared.	Back:<br>r = -0.01<br><br>Neck:<br>r = 0.26<br><br>Both combined:<br>r = -0.02	Y for both

BPI-IS Brief Pain Inventory Interference Subscale; LBP, low back pain; NDI, Neck Disability Index; RMDQ, Roland Morris Disability Questionnaire ;SF-12v2, short form of the Health Survey; VAS, visual analogue scale.

3 – Hypotheses for evaluating construct validity of a two-factor model

**Hypothesis**	**Justification**	**Result**	**Confirmed?**
**Physical domain**			
1. The BPI-IS physical domain will have at least a moderate positive correlation with the RMDQ in those with LBP.	The BPI-IS physical domain measures a similar but not identical construct to the RMDQ (physical interference from pain versus disability) therefore a moderate correlation is expected. This is more than what is expected for the affective domain where the expected correlation is at least weak. This is supported by literature which has found the BPI-IS correlates highly with the RMDQ (r=0.81) in a population with osteoarthritis and general non-cancer pain, but these are non-acute which is different from our acute LBP population.[34,35]	0.60	Yes
2. The BPI-IS physical domain will have at least a moderate positive correlation with the NDI in those with neck pain.	The BPI-IS physical domain measures a similar but not identical construct to the NDI (physical interference from pain versus disability from pain) therefore a moderate correlation is	0.58	Yes

	expected. This is more than what is expected for the affective domain where the expected correlation is at least weak. The NDI has been shown to correlate moderately with other PROMs measuring physical function/disability such as work and lifting in neck pain populations.[36]		
3. The BPI-IS physical domain will have at least a weak negative correlation with the SF-12v2 (Part 1) e.g. as SF-12v2 score increase (worse general health), BPI-IS scores decrease (more physical interference from pain).	Part 1 asks about general health. General health is a different construct to physical interference from pain however it is sensible to assume that the two constructs are somewhat similar i.e as physical interference from pain reduces, general health improves. Back and neck pain has been found to be associated with general health in a Danish twin study.[37] This is the same expected correlation as for the affective domain.	0.30	Yes
4. BPI-IS physical domain will have at least a strong negative correlation with	Part 2 asks about typical daily activities. This is a generic domain, and theoretically the same domain as the	0.09	No

the SF-12v2 (Part 2). As SF-12v2 score increase (improving ability to do daily activities), BPI-IS scores decrease (less physical interference from pain).	BPI-IS physical domain therefore we expect a strong correlation. This is stronger than what is expected for the affective domain where only a weak correlation is hypothesised. Both of these tools are not disease specific (unlike the NDI and RMDQ) which is why this correlation is expected to be stronger.<br>This has been demonstrated in literature regarding chronic pain and daily activities[38] and we would expect similar results with acute pain for the duration of the pain episode.		
5. BPI-IS physical domain will have at least a moderate negative correlation with the SF-12v2 (Part 3) e.g. as SF-12v2 score increase (improving ability to accomplish tasks) BPI-IS physical scores decrease	Part 3 asks about limitations of activities in the past week due to physical health, specially accomplishing less and being limited in kinds of activities. This is a similar construct to physical interference from pain therefore we expect at least a moderate correlation. The correlation	-0.10	No

(less physical interference from pain).	is expected to be weaker than that proposed in hypothesis 4 (above) because there may be more of an affective component to accomplishing tasks compared to being physically able to do tasks. It is expected to be stronger than the correlation with the affective domain (at least weak).<br>No literature is available on this matter to our knowledge.		
6. The BPI-IS physical domain will have at least a moderate negative correlation with the SF-12v2 (Part 5) e.g. as SF-12v2 score increase (improving ability do normal work) BPI-IS scores decrease (less physical interference from pain).	Part 5 asks about how much the pain has interfered with your normal work. This is a similar construct to physical interference from pain, however it includes other factors such as concentration, therefore we expect at least a moderate correlation. This is stronger than the expected correlation with the affective domain (at least weak).<br>The AIHW states that back problems are a significant cause of loss of productivity.[39]	-0.22	No

7. The BPI-IS physical domain will have less than a weak negative correlation with the SF-12v2 (Part 6) e.g. as SF-12v2 score increase (improving feelings of peace and energy) BPI-IS scores decrease (less physical interference from pain).	It is unclear how feelings and peace/energy relate to physical interference from pain. It is sensible to expect that there may be some relationship but it is not likely to reach the threshold for weak, moderate or strong, given that the domains are quite different. We expect the correlation to be weaker than with the affective domain where the hypothesis is at least a weak correlation.	0.12	No
8. The physical domain of the BPI-IS will have at least a weak positive correlation with the BPI Pain Severity subscale.	The BPI-IS physical domain measures a different construct to the Pain Severity subscale, but it would be sensible to assume the two correlate to a weak degree.<br>This is supported by literature examining the relationship between acute pain and disability.[42] This is the same as for the affective domain.	0.34	Yes
9. The physical domain of the BPI- IS will have at least a weak positive correlation	The BPI-IS measures a different construct to pain. However, it is sensible to assume that pain and	0.32	Yes

with the VAS pain scale (0-10) (average pain subscale of BPI-PI).	physical pain interference would correlate. This is the same as for the affective domain.<br>This is supported by literature showing a moderate to strong correlation between the entire BPI-IS and pain scales such as VAS and NRS in chronic LBP.[43] This population is acute, therefore the correlation may only be weak.		
10. The physical domain of the BPI-IS will not correlate with the location of a participant's pain (back, neck or both).	The location of pain (back vs neck) should not correlate with the physical interference from pain. This is the same as the affective domain.<br>Neither neck pain nor back pain has been demonstrated in literature to be more painful or more activity limiting than the other. This is the same as the affective domain.	-0.12	Yes
11. The physical domain of the BPI-IS will not correlate with gender.	There is no literature that suggests males or females experience more physical interference due to pain.<br>Therefore, we expect similar	-0.01	Yes

	distributions of scores within gender groups experiencing back and neck pain.		
**Affective domain**			
1. The BPI-IS affective domain will have at least a weak correlation with the RMDQ in those with LBP.	Affective interference from pain is likely related to the domain measured by the RMDQ (disability), but less so than the physical domain where we are expecting at least a moderate correlation. Psychological and mental wellbeing (similar domains to affect) have been shown in literature to correlate with measures of disability in chronic pain populations, so it may also correlate in this acute population.[44,45]	0.52	Yes
2. The BPI-IS affective domain will have at least a weak correlation with the NDI in those with neck pain.	Affective interference from pain is likely related to the domain measured by the NDI (disability), but less so than the physical domain where the expected correlation is at least moderate. Distress has been found to be associated with neck pain which is a somewhat similar domain to affect.[46]	0.64	Yes

3. The BPI-IS affective domain will have at least a weak negative correlation with the SF-12v2 (Part 1) e.g. as SF-12v2 score increase (worse general health), BPI-IS scores decrease (more affective interference from pain).	Part 1 asks about general health. General health is a different construct to affective interference from pain however it is sensible to assume that the two constructs are somewhat similar i.e. as general health improves, affective interference from pain reduces. Back and neck pain has been found to be associated with general health in a Danish twin study.[37] This is the same expected correlation as for the physical domain.	0.31	Yes
4. BPI-IS affective domain will have at least a weak negative correlation with the SF-12v2 (Part 2) As SF-12v2 score increase (improving ability to do daily activities), BPI-IS scores decrease (less affective interference from pain).	Part 2 asks about typical daily activities. While we expect this to be strongly correlated with the physical domain, there is much less evidence of a relationship between affect and ability to do daily activities. It is sensible to expect at least a weak correlation as having less ability to do daily activities is likely to be accompanied by reduced affect.	0.07	No

5. BPI-IS affective domain will have at least a weak negative correlation with the SF-12v2 (Part 3) e.g. as SF-12v2 score increase (improving ability to accomplish tasks) BPI-IS scores decrease (less affective interference from pain).	Part 3 asks about limitations of activities in the past week due to physical health, specially accomplishing less and being limited in kinds of activities. It is sensible to expect at least a weak correlation as having less ability to accomplish tasks is likely to be associated with reduced affect, but less so than the physical domain where the correlated is expected to be at least moderate.	-0.07	No
6. The BPI-IS affective domain will have at least a weak negative correlation with the SF-12v2 (Part 5) e.g. as SF-12v2 score increase (improving ability do normal work) BPI-IS scores decrease (less affective interference from pain).	There is a lack of evidence for a direct association between ability to work and affect, but it is sensible to expect at least a weak correlation as having less ability to work is likely to be associated with reduced affect, but less so then the physical domain where the expected correlation is at least moderate.	-0.14	No

7. The BPI-IS affective domain will have at least a weak negative correlation with the SF-12v2 (Part 6) e.g. as SF-12v2 score increase (improving feelings of peace and energy) BPI-IS scores decrease (less affective interference from pain).	The relationship between the domains of peace/energy and affect but have not been studied. It is sensible to expect a weak correlation due to the overlap of the domains, however we are expecting it to be weaker than that with the physical domain where the expected correlation is less than weak (0.0 to 0.29).	-0.09	No
8. The affective domain of the BPI-IS will have at least a weak positive correlation with the BPI Pain Severity subscale.	Pain and mood (affect) have been shown to be related but have a complex relationship, especially in acute conditions where pain and any mood changes are transient.[47] Therefore, we are expecting a weak correlation. This is the same as for the physical domain.	0.34	Yes
9. The affective domain of the BPI- IS will have at least a weak positive correlation with the VAS pain scale (0-	The BPI-IS affective domain measures a different construct to pain. However, it is sensible to assume that pain and affective interference from pain would	0.31	Yes

10) (average pain subscale of BPI-PI).	correlate. This is the same as for the physical domain.<br>This is supported by literature showing a moderate to strong correlation between the whole BPI-IS and pain scales such as VAS and NRS in chronic LBP [43]. As our population is acute, the correlation may only be weak.		
10. The affective domain of the BPI-IS will not correlate with the location of a participant's pain (back, neck or both).	The location of pain (back vs neck) should not correlate with the affective interference from pain.<br>Neither neck pain nor back pain has been demonstrated in literature to be more emotionally distressing than the other. This is the same as the physical domain.	0.05	Yes
11. The physical domain of the BPI-IS will not correlate with gender.	There is no literature that suggests males or females experience more affective interference due to pain.<br>Therefore, we expect similar distributions of scores within gender groups experiencing back and neck	0.01	Yes

	pain. This is the same as the physical domain.		

BPI-IS Brief Pain Inventory Interference Subscale; LBP, low back pain; NDI, Neck Disability Index; RMDQ, Roland Morris Disability Questionnaire ;SF-12v2, short form of the Health Survey; VAS, visual analogue scale.

*All hypotheses were tested using Pearson's correlation coefficient to measure convergent validity.

4 – Summary of Global Perceived Effect score (as reported in manuscript Tables 4 and 5) correlation coefficients by pain location

<u>**One factor**</u>	n	Correlation coefficient
Back	196	-0.35
Neck	25	-0.21
Both	19	-0.40
All combined	240	-0.35
<u>**Two factor**</u>		
**Physical**		
Back	196	-0.37
Neck	25	-0.20
Both	19	-0.42
All combined	240	-0.37
**Affective**		
Back	196	-0.29
Neck	25	-0.20
Both	19	-0.34
All combined	240	-0.48
